# Animal efficacy study of a plant extract complex (BEN815) as a potential treatment for COVID-19

**DOI:** 10.1371/journal.pone.0291537

**Published:** 2023-09-14

**Authors:** Moon Ho Do, Hua Li, Su Yeon Cho, Subin Oh, Ju Hwan Jeong, Min-Suk Song, Jong-Moon Jeong

**Affiliations:** 1 Biotechnology Research Center, Ben’s Lab Co., Ltd., Anyang-si, Gyeonggi-do, Republic of Korea; 2 Department of Microbiology, Chungbuk National University College of Medicine and Medical Research Institute, Cheongju-si, Chungcheongbuk-do, Republic of Korea; 3 Department of Bioscience, The University of Suwon, Hwasung-si, Gyeonggi-do, Republic of Korea; Concordia University, CANADA

## Abstract

In a short time, several types of injectable and oral therapeutics have been developed and used to effectively manage patients with coronavirus disease 2019 (COVID-19). BEN815 is an improved mixture of three extracts (*Psidium guajava*, *Camellia sinensis*, and *Rosa hybrida*) recognized by the Ministry of Food and Drug Safety of Korea as a health food ingredient that alleviates allergic rhinitis. The current animal efficacy study was performed to assess its probability of improving COVID-19 symptoms. BEN815 treatment significantly increased the survival of K18-hACE2 transgenic mice and reduced viral titers in the lungs at 5 days post infection (DPI). Furthermore, the lungs of the treated mice showed mild tissue damage at 5 DPI and nearly complete recovery from COVID-19 at 14 DPI. BEN815 appears to be an effective and minimally toxic anti-SARS-CoV-2 agent in mice and has potential for clinical applications.

## Introduction

The coronavirus disease 2019 (COVID-19) pandemic resulted from the global spread of the novel severe acute respiratory syndrome coronavirus 2 (SARS-CoV-2) in December 2019, posing a serious threat to public health. Infection with this virus is accompanied by symptoms ranging from mild—fever, cough, and anosmia—to severe—multiorgan dysfunction, acute respiratory distress syndrome, sepsis, secondary infection, and severe pneumonia [1, 2]. In a relentless effort to end the pandemic, effective vaccines against SARS-CoV-2 infection were rapidly developed. However, owing to the limited number of medications that can manage this infection, developing a therapeutic agent to alleviate COVID-19 symptoms is urgently needed.

SARS-CoV-2 attaches to target cells via the binding of its spike protein to the host cell angiotensin-converting enzyme (ACE) 2 receptor [3], causing an uncontrolled inflammatory response [4]. ACE2 belongs to the renin–angiotensin system and is a host tissue protective factor. ACE2 levels are downregulated by interacting with the SARS-CoV-2 virus in the lungs, leading to severe COVID-19 symptoms [5]. SARS-CoV-2 infection also triggers the secretion of numerous cytokines and chemokines, including interleukin (IL)-1, IL-2, IL-4, IL-6, IL-7, IL-10, interferon-γ, monocyte chemoattractant protein-1, macrophage inflammatory protein-1A, and tumor necrosis factor-α [6]. SARS-CoV-2 infection and increased levels of inflammatory cytokines can reduce ACE2 levels and trigger a cytokine storm associated with disease severity [7]. Therefore, an appropriate combination of antiviral and anti-inflammatory agents may represent an effective therapeutic strategy.

BEN815 is an herbal mixture comprising extracts of green tea (*Camellia sinensis*) leaves, guava (*Psidium guajava*) leaves, and rose (*Rosa hybrida*) petals. It is an improvement of the BENDU381 formulation ratio, a formulation of PEM381 designed for oral administration to humans. BENDU381 has been previously reported to alleviate allergic inflammation [8–10] and is registered as an individual recognition-type health food ingredient to alleviate allergic rhinitis by the Ministry of Food and Drug Safety of the Republic of Korea (No. 2009–16). Various antiviral agents that directly act on viruses are under development or being assessed in clinical trials. They target RNA-dependent RNA polymerase (RdRp), main protease (M^pro^, also known as 3CL^pro^), and papain-like protease (PL^pro^) [11]. *In silico* structural analyses suggested that epigallocatechin gallate (EGCG) from green tea, an active compound of BEN815, inhibits the infectious activity of SARS-CoV-2 by binding to the RdRp active site (based on molecular docking studies) [12]. Furthermore, EGCG inhibits SARS-CoV-2 Mpro and Nsp15 activities *in vitro* [13, 14]. In addition, we previously showed that BEN815 has high antiviral efficacy against SARS-CoV-2 and increases the survival of mice with lipopolysaccharide-induced hyperinflammation by reducing inflammatory cytokine levels [15]. However, BEN815 has not been shown to effectively treat COVID-19 symptoms. Hence, in the current study, we employed a mouse model to determine the potential of BEN815 as a new COVID-19 therapeutic agent.

## Materials and methods

### BEN815 and nirmatrelvir preparation

Guava leaf, green tea leaf, and rose petal extracts were purchased from Daejong Biotech Co., Ltd. (Seoul, Korea). We then prepared BEN815 by mixing these extracts in a 65:30:5 ratio, respectively. The active compounds were quercetin, EGCG, ellagic acid, gallic acid, kaempferol, and myricetin, the content and efficacy of which were analyzed in a previous study [15]. The mixture was dissolved in 0.9% saline and stored at -20°C. Nirmatrelvir was obtained from MedChemExpress (Monmouth Junction, NJ, USA); a stock solution was prepared by dissolving nirmatrelvir in 0.9% saline containing 1% dimethyl sulfoxide and 20% sulfobutylether-β-cyclodextrin (MedChemExpress), which was stored at -20°C.

### Model organisms and transfection

Eight-week-old female K18-hACE2 transgenic mice (B6.Cg-Tg(K18-ACE2)2Prlmn/J) were purchased from Orient Bio Inc. (Seongnam, Korea), provided *ad libitum* access to standard chow diet and water, and maintained under a 12-h light/dark cycle at a temperature of 22 ± 2°C and a relative humidity of 55 ± 5%. All procedures were approved by the Institutional Animal Care and Use Committee of Chungbuk National University (CBNUA-1659-22-01). All experiments were conducted by trained researchers in a biosafety level 3 facility, following the approved standard operating protocols by the Institutional Biosafety Committee of Chungbuk National University. After 8 days of acclimatization, the animals were randomly allocated into six experimental groups (uninfected, uninfected-BEN815, SARS-CoV-2, Nirmatrelvir, BEN815-200, and BEN815-300 groups), with five mice in the uninfected control group and 10 mice in all other groups (N = 55).

**Fig 1:**
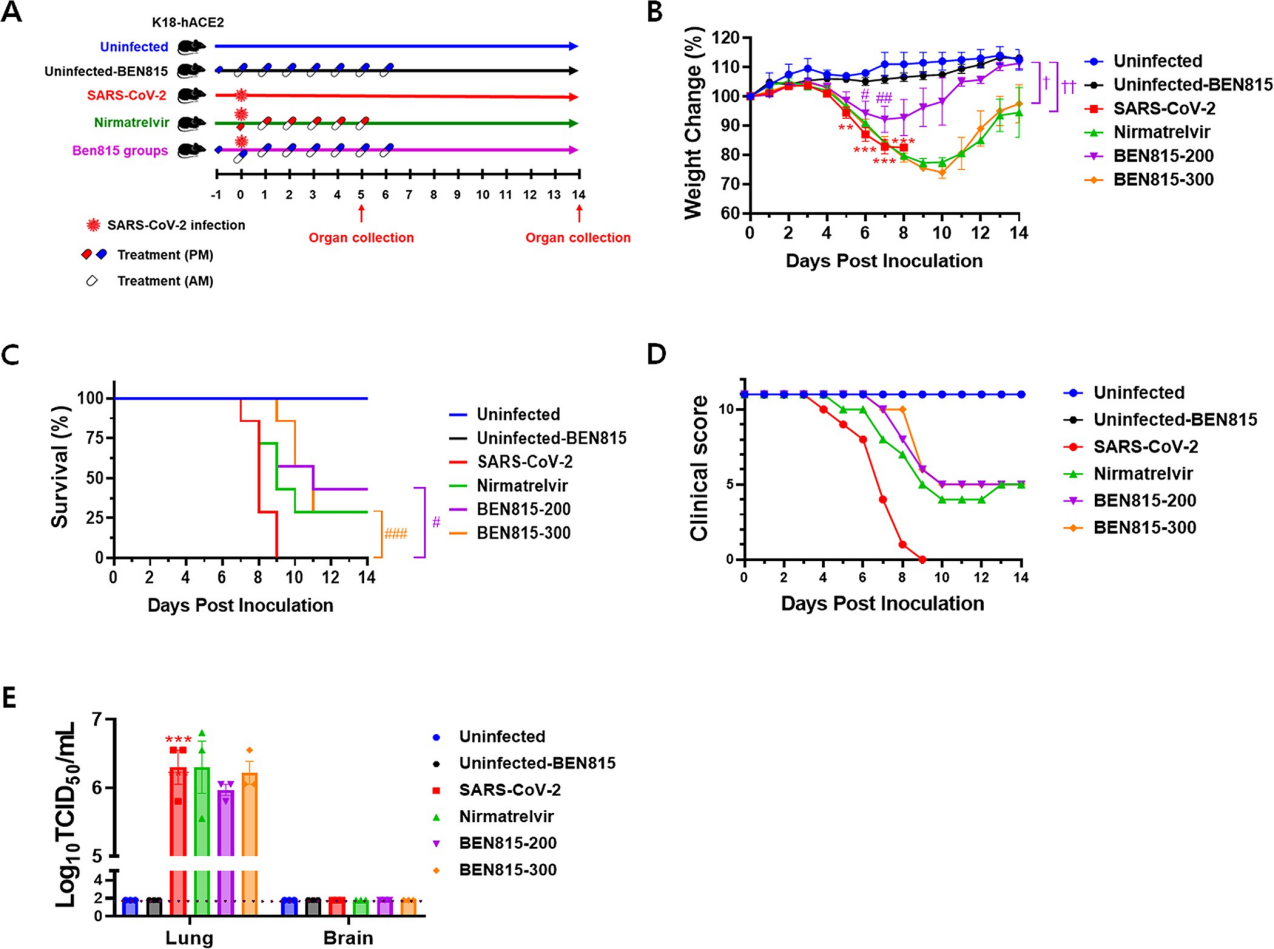
Therapeutic efficacy of BEN815 in SARS-CoV-2-infected K18-hACE2 transgenic mice. (A) Schematic illustration of experimental design. (B) Changes in body weight are expressed as a percentage of body weight on day 0 for the 14-day monitoring (one-way ANOVA with Tukey’s test; **p < 0.01 and ***p < 0.001 vs. the uninfected group, ^#^p < 0.05 and ^###^p < 0.01 vs. the SARS-CoV-2 group, and ^$^p < 0.05 and ^$ $^p < 0.01 vs. the BEN815-200 group). (C) Kaplan–Meier survival curves (log-rank [Mantel–Cox] test; ^#^p < 0.05 and ^###^p < 0.001 vs. the SARS-CoV-2 group). (D) Clinical score for appearance (fur, eyes closure), activity level, and movement during the test period expressed as cumulative clinical scores of mice in each group. (E) Infectious SARS-CoV- titers were estimated using lung and brain tissues collected at 5 DPI. The viral titers were expressed as log_10_TCID_50_/mL, and the limit of detection is indicated by a dashed line. Statistical significance was calculated using one-way ANOVA with Tukey’s multiple comparison test; ***p < 0.001 vs. the uninfected group.

For SARS-CoV-2 infection, we anesthetized the mice via isoflurane inhalation and intranasally inoculated them with 50 μL of the viral solution containing 3.7 Log_10_TCID_50_/50 μL (5MLD_50_) of BetaCoV/Korea/KCDC03/2020 (NCCP43326) virus, isolated by the National Culture Collection for Pathogens of the Korea Center for Disease Control and Prevention. To the BEN815 groups, BEN815 was orally administered at 200 mg/kg (BEN815-200) or 300 mg/kg (BEN815-300) 24 h before infection (Fig 1A). Subsequently, the administration was continued every 12 h until 6 days post infection (DPI) (total 15 times, daily dose: 400 or 600 mg/kg). Simultaneously, we administered BEN815 (300 mg/kg) to the negative test group (uninfected-BEN815). Meanwhile, the nirmatrelvir groups were orally administered 10 mg/kg of nirmatrelvir 6 h post infection and every 12 h thereafter until 5 DPI (11 times in total, daily dose: 20 mg/kg). The uninfected and untreated SARS-CoV-2-infected mice were administered saline. At 5 DPI, we euthanized three animals per group using isoflurane and collected lung and brain tissues. The tissues were stored at -80°C for viral titer assay, histopathology, and immunohistochemistry. The clinical symptoms, weight changes and survival rate of the mice were recorded up to 14 DPI. The clinical scoring system comprised several categories, including body weight, appearance (fur condition, eye closure), activity level, and movement. These parameters were evaluated following standard guidelines, and the scoring scale ranged from 0 to 11, with 11 representing the highest achievable score [16]. We then euthanized all surviving mice at the end of the experiment and collected their lung and brain tissues. Humane euthanasia was performed via CO_2_ inhalation when the mice lost more than 25% of their body weight at 0 DPI. Animals that died before meeting the euthanasia criteria were immediately necropsied.

### Cell culture and viral titer assay

Vero E6 cells (VERO C1008 (Vero 76, clone E6, Vero E6)) were obtained from the American Type Culture Collection (CRL1586, Rockville, MD, USA) and cultured in DMEM (Gibco, Gaithersburg, MA, USA) supplemented with 10% fetal bovine serum and 1% antibiotic–antimycotic solution at 37°C in a 5% CO_2_ incubator (Sanyo Co., Osaka, Japan).

To determine the viral titer, we homogenized frozen tissues with 500 μL of serum-free DMEM using a Tissue Lyser II (Qiagen, Hilden, Germany) and serially diluted the supernatants 10-fold in serum-free DMEM. Subsequently, we seeded Vero E6 cells in 96-well culture plates (1 × 10^4^ cells/well) and incubated them for 24 h. The cells were then inoculated with serially diluted samples for 1 h. After inoculation, we replaced the inoculum with fresh serum-free DMEM. After 96 h, we stained the cells with 10% crystal violet solution and calculated the 50% tissue culture infective dose (TCID_50_) using the Reed–Muench method [17].

### Histology and immunohistochemistry

We fixed the mouse lung and brain tissues in 10% formalin solution (Sigma Aldrich, St. Louis, MO, USA) and then paraffin-embedded and sectioned them at 3 μm thickness. Subsequently, we stained the sectioned slides with hematoxylin and eosin (H&E) to assess histopathological changes. To observe the SARS-CoV-2 antigen, we performed chromogenic immunohistochemical staining using the rabbit anti-SARS-CoV-2 (2019-nCoV) nucleocapsid antibody (40143-R019, SinoBiological, Beijing, China) and OmniMap anti-Rb HRP secondary antibody (760–4311, Roche Diagnostics, Indianapolis, IN, USA). Immunohistochemical staining was processed using an automated system, the Ventana Discovery Ultra (Roche Diagnostics); all slides were visualized using a Panoramic 250 Flash III slide scanner (3DHISTECH Ltd., Budapest, Hungary) and evaluated using CaseViewer 2.4 software (3DHISTECH Ltd.).

### Statistical analysis

We expressed all results as the mean ± standard error of the mean (SEM) and applied a one-way analysis of variance to determine the statistical significance between groups followed by Tukey’s test. Additionally, we described the survival curves using the Kaplan–Meier method with the log-rank (Mantel–Cox) test. Statistical analyses were performed using GraphPad Prism software version 9 (San Diego, CA, USA), and statistical significance was set at P < 0.05.

## Results

### Therapeutic efficacy of BEN815 with respect to mortality and viral titers

We initiated BEN815 treatment 24 h prior to infection and administered it twice daily for 6 consecutive days (Fig 1A). Six hours after infection, we began treatment with nirmatrelvir, a positive control, twice daily for five consecutive days. As expected, at 5 DPI, body weight began to decrease, and the SARS-CoV-2 infection group lost 17.9% body weight at 8 DPI and did not survive beyond 9 DPI (Fig 1B and 1C). The BEN815-200 group experienced maximum weight loss (8.0%) at 7 DPI, which was 9.9% higher than that in the SARS-CoV-2 group. However, the nirmatrelvir and BEN815-300 groups lost 22.5% and 25.9% body weight, respectively, at 9 or 10 DPI, showing no protective effect against weight loss induced by SARS-CoV-2 infection. Moreover, the BEN815-200 group showed the highest survival rate at 42.9%, higher than that of the nirmatrelvir group (28.9%). Additionally, the BEN815-300 group showed the highest survival rate until 8 DPI; however, death occurred 9 DPI. Hence, the final survival rate was the same as that of the nirmatrelvir group.

Regarding the clinical features of SARS-CoV-2 infection following BEN815 administration, the clinical score of the BEN815-200 and BEN815-300 groups decreased to 5 points from 7 to 10 DPI, however, it remained at this level thereafter (Fig 1D). Meanwhile, the nirmatrelvir group exhibited a decrease at 5 DPI and peaked at 10 DPI, recovering slightly at 13 DPI with a score equal to that of the BEN815 treatment groups.

To evaluate the protective effect of BEN815 on mouse mortality, we measured the viral titers. Studies have shown that high viral titers and infectious viruses can be detected in the lungs of K18-hACE2 transgenic mice with SARS-CoV-2 infection [4]. Similarly, the same mouse model of SARS-CoV-2 infection showed high viral titers in the brain during nasal infection [18]. Therefore, we sought to confirm whether the virus was present in the tissues of lungs and brain collected 5 DPI (Fig 1E). Titers in the lung tissue of SARS-CoV-2-infected mice were measured at 6.301 Log_10_TCID_50_/mL. The average viral titer in the lungs of the nirmatrelvir-treated group was 6.301 Log_10_TCID_50_/mL, relatively the same as that of the infected group. The average viral titer in the lung tissue of the BEN815 200 mg/kg group was 5.967 Log_10_TCID_50_/mL, which did not differ significantly from that of the SARS-CoV-2 group; however, it was 2.15-fold lower than that in the SARS-CoV-2 infection only and nirmatrelvir groups. An average of 6.218 Log_10_TCID_50_/mL was measured in the BEN815 300 mg/kg group, which was only 1.21 times lower than in the SARS-CoV-2-infected group. No viral infection was observed in the brain.

### Therapeutic efficacy of BEN815 with respect to histological changes

**Fig 2:**
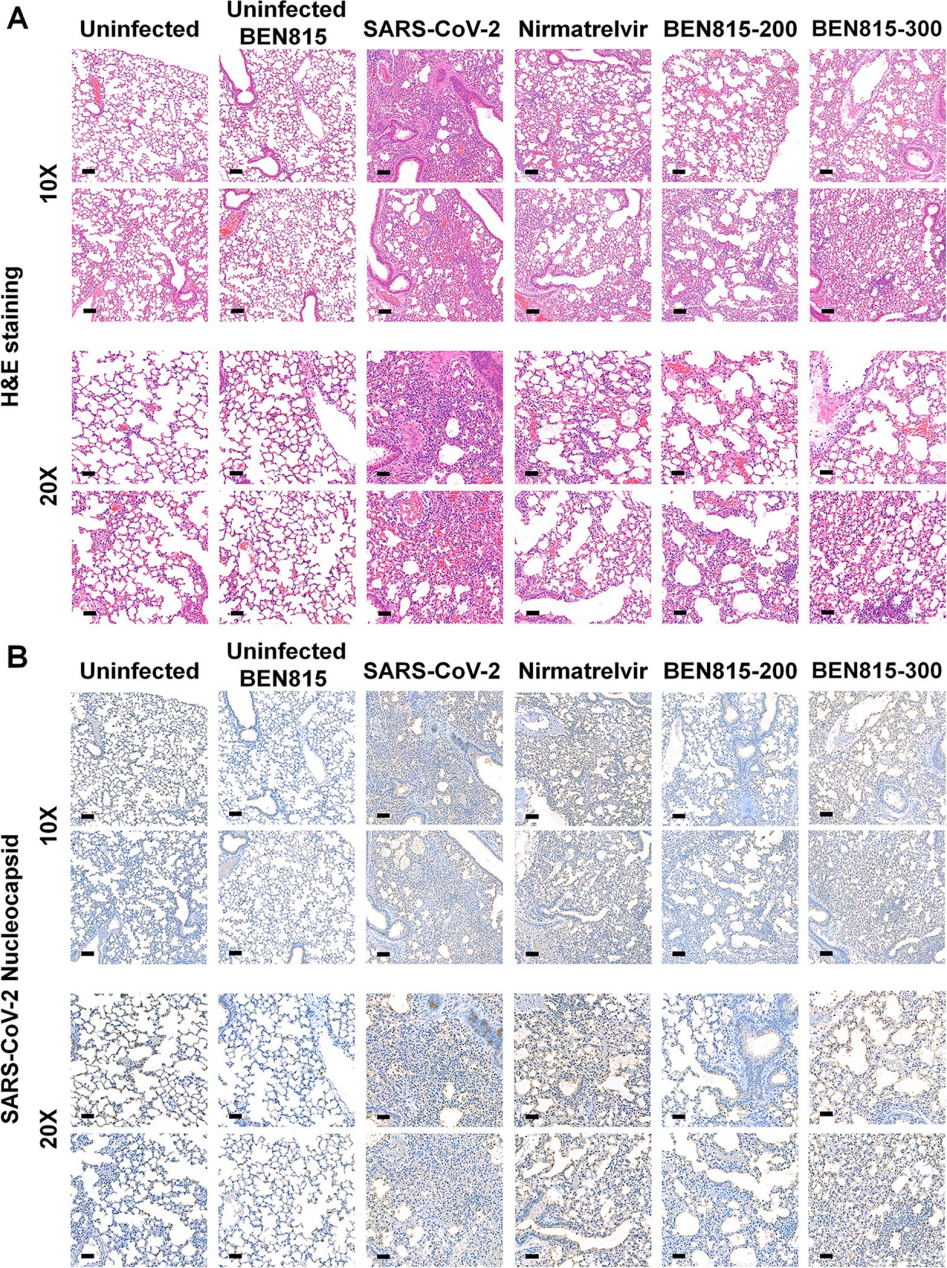
Histopathological and immunohistochemical analyses of K18-hACE2 transgenic mouse tissues after SARS-CoV-2 infection at 5 DPI. Representative hematoxylin and eosin (H&E) and immunohistochemical staining with an anti-nucleocapsid antibody of lung tissue. Images are shown at low (10×) and high (20×) power magnifications. Scale bars represent 50 μm at 10× magnification and 100 μm at 20× magnification.

To evaluate the protective effect of BEN815 histologically, we performed histopathological examination and immunohistochemistry (IHC) of the lungs and brain. H&E staining of lung tissue from the SARS-CoV-2 group collected at 5 DPI revealed severe lesions, including many immune and destroyed cells, along with the development of pneumonia (Fig 2). In addition, many immunostained SARS-CoV-2 proteins were detected in the lung tissue. The nirmatrelvir and BEN815 300 groups developed pneumonia in the lung tissue at a similar or slightly alleviated level compared to that of the SARS-CoV-2 group, and SARS-CoV-2 antigens were observed over a wide area. However, in the BEN815 200 group, lower levels of inflammation and mild tissue damage were observed compared to that in the SARS-CoV-2 group in H&E staining, and a low level of SARS-CoV-2 antigens was detected by IHC staining. In the uninfected-BEN815 group, tissue morphology similar to that of the uninfected group was observed; therefore, it was expected that no toxicity was elicited by BEN815. As in the viral titer analysis, no histological changes in the brain were detected from SARS-CoV-2 infection (S1 Fig).

**Fig 3:**
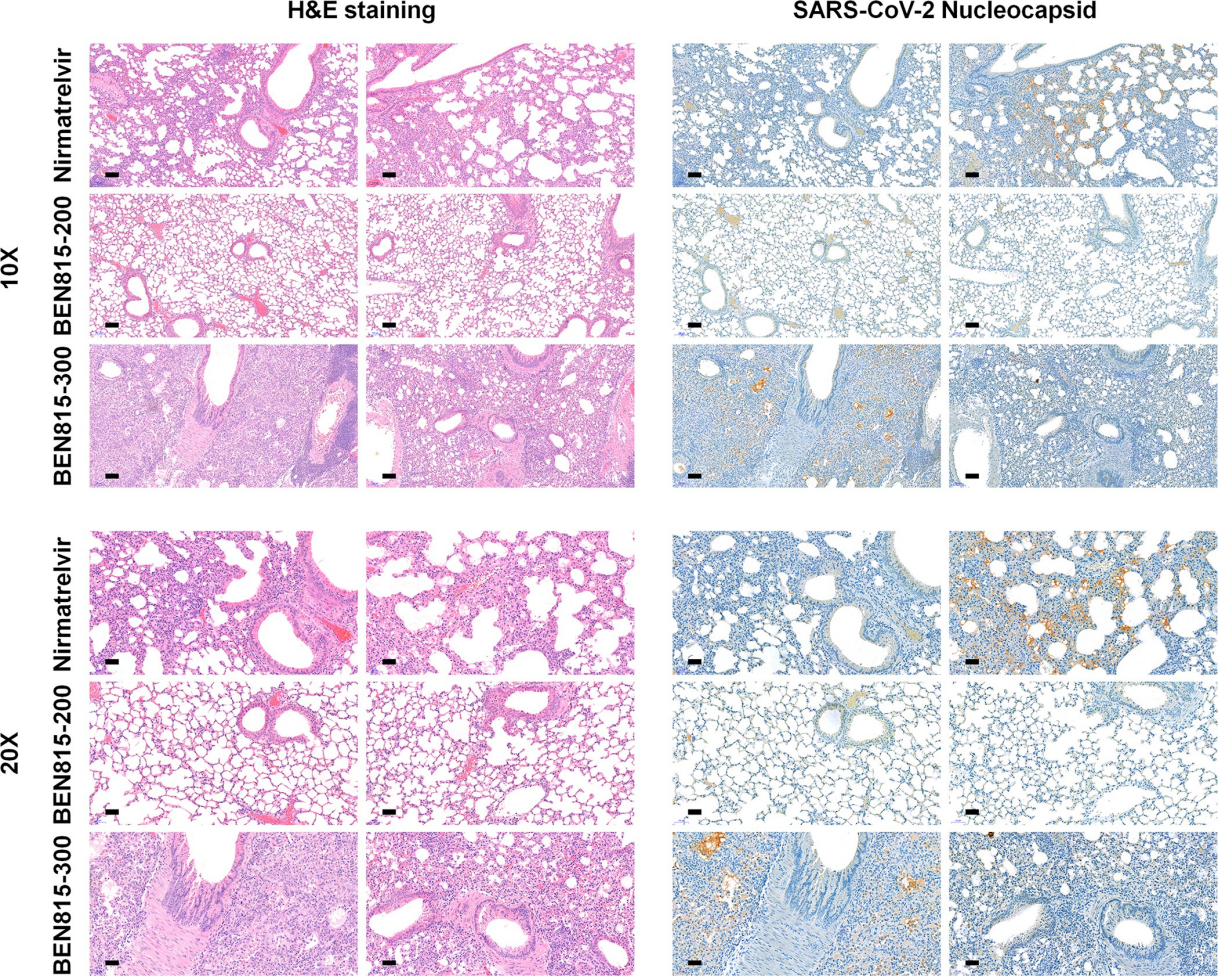
Histopathological and immunohistochemical analyses in K18-hACE2 transgenic mouse tissues after SARS-CoV-2 infection at 14 DPI. (A) Representative hematoxylin and eosin (H&E) and immunohistochemical staining with an anti-nucleocapsid antibody of lung tissue. Images are shown at low (10×) and high (20×) power magnifications. Scale bars represent 50 μm at 10× magnification and 100 μm at 20× magnification.

To confirm the degree of tissue improvement and residual virus, surviving mice were euthanized at 14 DPI, and lung and brain tissues were collected. Lung tissue damage and multiple residual SARS-CoV-2 proteins were observed in nirmatrelvir-treated mice (Fig 3). Similarly, many SARS-CoV-2 antigens and pneumonia were detected in mice from the BEN815 300 group. However, at 14 DPI, tissues from the mice in the BEN815 200 group showed considerable lung tissue recovery compared to those collected 5 DPI—inflammation sites could not be observed, and SARS-CoV-2 antigen was not detected in the lung tissue. Therefore, it was deemed that the lung tissue had recovered to average levels. These results suggest that the optimal dose of BEN815 in mice is 200 mg/kg bid (twice daily). At 5 DPI, no histological changes or virus distribution were observed in the brain in any groups (S2 Fig). Collectively, these findings suggest that BEN815 is effective in reducing the burden of SARS-CoV-2 and has the potential to provide a survival advantage over one of the most widely used therapeutics, nirmatrelvir.

## Discussion

Despite efforts to prevent SARS-CoV-2 infection through the development of various vaccines, the uncontrolled COVID-19 pandemic and emergence of SARS-CoV-2 variants continue to drive the development of effective antiviral therapeutics to target SARS-CoV-2 and alleviate symptoms of the infection. Currently approved antiviral treatments include remdesivir, molnupiravir, and nirmatrelvir. Remdesivir, which targets RdRp and was the first drug authorized for emergency use as a treatment for COVID-19, is being used to treat COVID-19 patients in many countries [19]. However, recent clinical studies have highlighted its lack of efficacy [20] and serious side effects, such as nausea, vomiting, respiratory failure, elevated liver enzyme levels, hypokalemia, and kidney injury [21, 22]. Another drug approved for emergency use as an RdRp target and the first oral antiviral for COVID-19, molnupiravir, has shown clinical benefits in reducing hospitalizations and deaths in patients not hospitalized for COVID-19 [23]. Nonetheless, it has not demonstrated clinical benefits in patients hospitalized with COVID-19 [24]. Although no short-term serious side effects have been reported, its use is limited in pregnant women and men of childbearing potential who have sexual intercourse with women of childbearing potential, owing to the risks associated with toxicity to embryo-fetuses and offspring [25]. Nirmatrelvir interferes with the M^pro^ of SARS-CoV-2. Together with ritonavir, it is an essential component of Paxlovid, and can prevent 89% of hospitalizations and deaths when taken within 3 days of symptom onset [26]. However, since Paxlovid acts by inhibiting CYP3A, it is difficult to use in patients taking medicines that may affect metabolic processes or induce the breakdown of nirmatrelvir or ritonavir [27]. Furthermore, it can induce drug resistance to HIV-1 [28], and recently, a study reported that the substitution of SARS-CoV-2 M^pro^ can weaken binding to nirmatrelvir, leading to resistance [29]. Furthermore, the high prices of these drugs can significantly hinder their application. Thus, safe therapeutic agents that can improve the survival rate and symptoms of SARS-CoV-2 infection without these limitations are urgently needed.

To resolve the adverse events, exceptions in select groups of patients, and cost limitations outlined above, the efficacy of BEN815 was evaluated in this study to determine the protective effects of BEN815 on SARS-CoV-2 as well as its comparable activity to previously developed antiviral agents using nirmatrelvir as a control. Considering that SARS-CoV-2 specifically binds to human ACE2, not to murine ACE2 [30], a transgenic murine model expressing K18-hACE2 was developed [31] and is widely used as an efficacy evaluation model for developing COVID-19 treatments [32–34]. In this study, SARS-CoV-2-inoculated animals rapidly lost weight by 5 DPI and began to die at 7 DPI. High levels of infectious virus and tissue damage were observed in the lungs of infected mice at 5 DPI. However, when BEN815 was administered at 200 mg/kg bid, the weight loss and mortality induced by infection were significantly reduced, along with improvement in clinical scores. Although the viral titer assay showed only slight improvement, histopathological analysis revealed low levels of pneumonia and coronavirus antigen in the tissue at 5 DPI; levels similar to those of normal tissues were observed in the recovered individuals at 14 DPI. Hence, 200 mg/kg BEN815 may exhibit excellent recovery ability and relieve COVID-19 symptoms. In addition, these results were superior to those of nirmatrelvir with respect to weight loss, viral titer, lung pathology, and virus-induced death; however, there were no differences in the clinical scores. Accordingly, it is expected to have positive therapeutic effects in clinical practice. However, in mice treated with 300 mg/kg bid, the survival rate and weight recovery were similar to those of the nirmatrelvir group, whereas the virus titer was similar to that of the SARS-CoV-2 group. Histopathological analysis of this treatment group also showed insignificant differences; therefore, the protective effects at this concentration were worse than those of the 200 mg/kg treatment group. The reasons for these results may vary; however, we speculate one possibility. That is, quercetin, known to have a potent anti-inflammatory effect [35], and EGCG, known to have excellent antiviral and anti-inflammatory efficacy [36, 37], account for high proportions of the content of BEN815, at 30.8% and 16.5%, respectively [15]. Therefore, at a high concentration of 300 mg/kg, the antiviral immunity of the host may have been reduced owing to the over-suppression of the antiviral immune response by the anti-inflammatory agent, thus rendering the initial viral suppression ineffective. The results of clinical trials with a large number of patients also found that administering strong immunosuppressants, such as glucocorticoids, during the early symptomatic stage increases the risk of infection [38]. Moreover, although quercetin can reportedly inhibit the 3CL^pro^ and PL^pro^ activity of SARS-CoV-2 by reducing the docking binding energy [39], in our previous study, quercetin did not exhibit antiviral activity in Vero E6 cells, and the antiviral activity of BEN815 was similar to that of EGCG [15]. Therefore, it is considered that quercetin does not have antiviral activity, and excessive intake of BEN815 might suppress antiviral efficacy by eliciting a strong anti-inflammatory effect due to the relatively high quercetin content. Thus, administering BEN815 at appropriate concentrations is crucial when attempting to alleviate SARS-CoV-2 symptoms, and balancing its antiviral and anti-inflammatory effects may affect efficacy. Although further studies are required, based on the results of a previous study [15] and this study, we can deduce that the optimal administration of BEN815 in mice is 400 mg/kg per day.

The overall findings of this study are encouraging for the development of new therapeutics; however, there are some limitations. We were not able to confirm the mechanisms underlying the action of BEN815 *in vivo* or the target (e.g., RdRp, M^pro^, or PL^pro^) responsible for its antiviral activity. Accordingly, further research is needed to determine the mechanisms underlying the effects of BEN815 and its targets to improve its antiviral activity against SARS-CoV-2 infection. However, its constituents, consumed as foodstuffs, have significant implications in reducing mortality and alleviating pneumonia in mice infected with SARS-CoV-2. Owing to these advantages, we expect to be able to conduct clinical trials for the development of new BEN815-based therapeutic agents in the future.

## Conclusions

In this study, we confirmed that BEN815 greatly increased the survival rate of mice infected with the SARS-CoV-2 virus. Additionally, BEN815 induced marked recovery of lung tissue, with no observed inflammatory sites or detected SARS-CoV-2 antigen in the lung tissue. Hence, BEN815 can effectively alleviate COVID-19 symptoms and holds potential as a novel therapeutic agent.

## Supporting information

S1 Fig Histopathological and immunohistochemical analyses of K18-hACE2 transgenic mouse tissues after SARS-CoV-2 infection at 5 DPI. H&E staining and immunohistochemical staining with an anti-nucleocapsid antibody of brain tissue. Images are shown at low (10×) and high (20×) power magnifications. Scale bars represent 50 μm at 10× magnification and 100 μm at 20× magnification.
(TIF)

S2 Fig Histopathological and immunohistochemical analyses in K18-hACE2 transgenic mouse tissues after SARS-CoV-2-infection at 14 DPI. H&E and immunohistochemical staining with an anti-nucleocapsid antibody of brain tissue. Images are shown at low (10×) and high (20×) power magnifications. Scale bars represent 50 μm at 10× magnification and 100 μm at 20× magnification.
(TIF)
