# Supplementary figures and images for: Animal efficacy study of a plant extract complex (BEN815) as a potential treatment for COVID-19
Source: PLoS One. 2023 Sep 14;18(9):e0291537. doi: 10.1371/journal.pone.0291537 (PMC10501575; doi:10.1371/journal.pone.0291537)

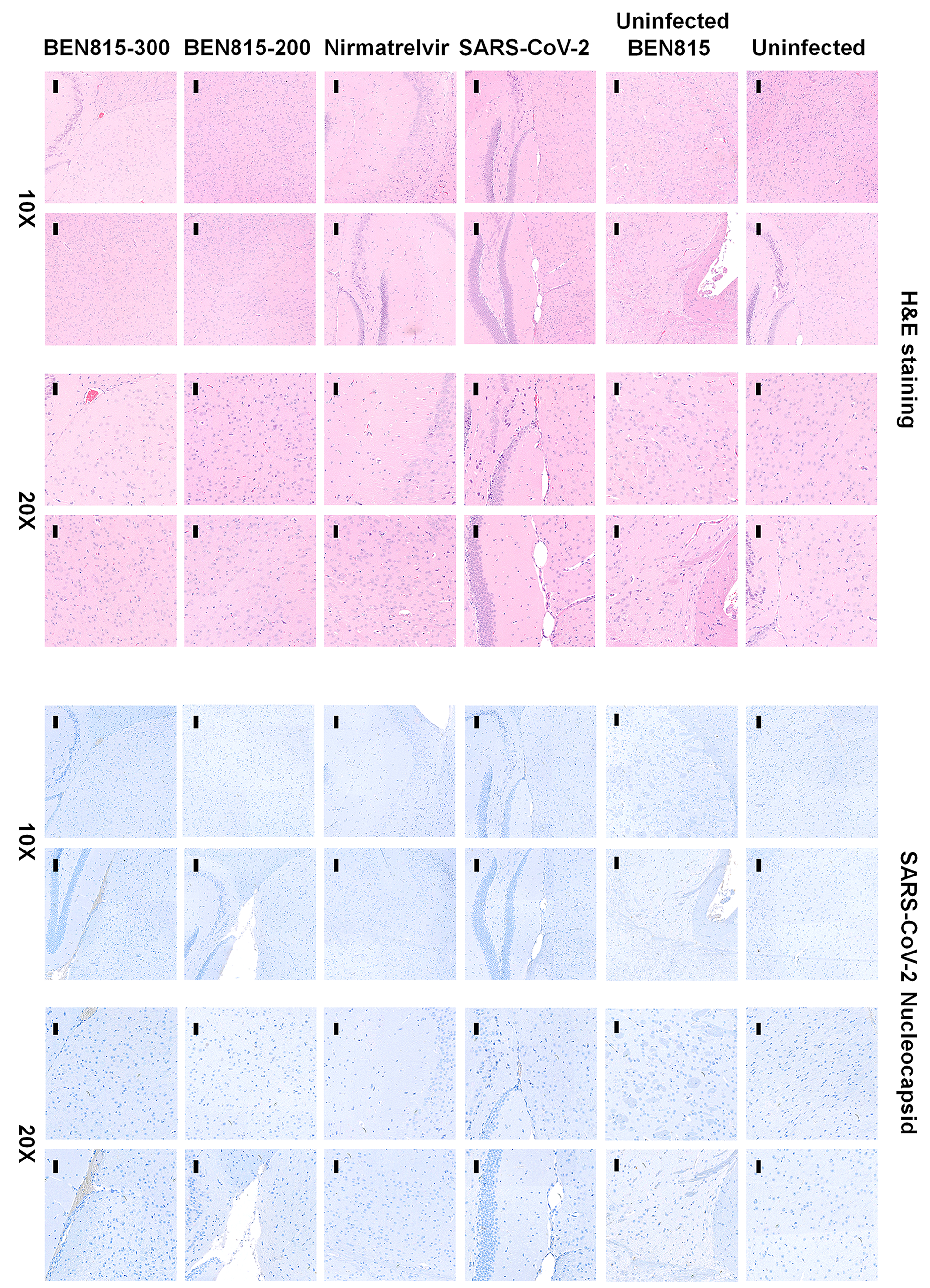

Supplement: S1 Fig — H&E staining and immunohistochemical staining with an anti-nucleocapsid antibody of brain tissue. Images are shown at low (10×) and high (20×) power magnifications. Scale bars represent 50 μm at 10× magnification and 100 μm at 20× magnification. (TIF) [file pone.0291537.s001.tif]

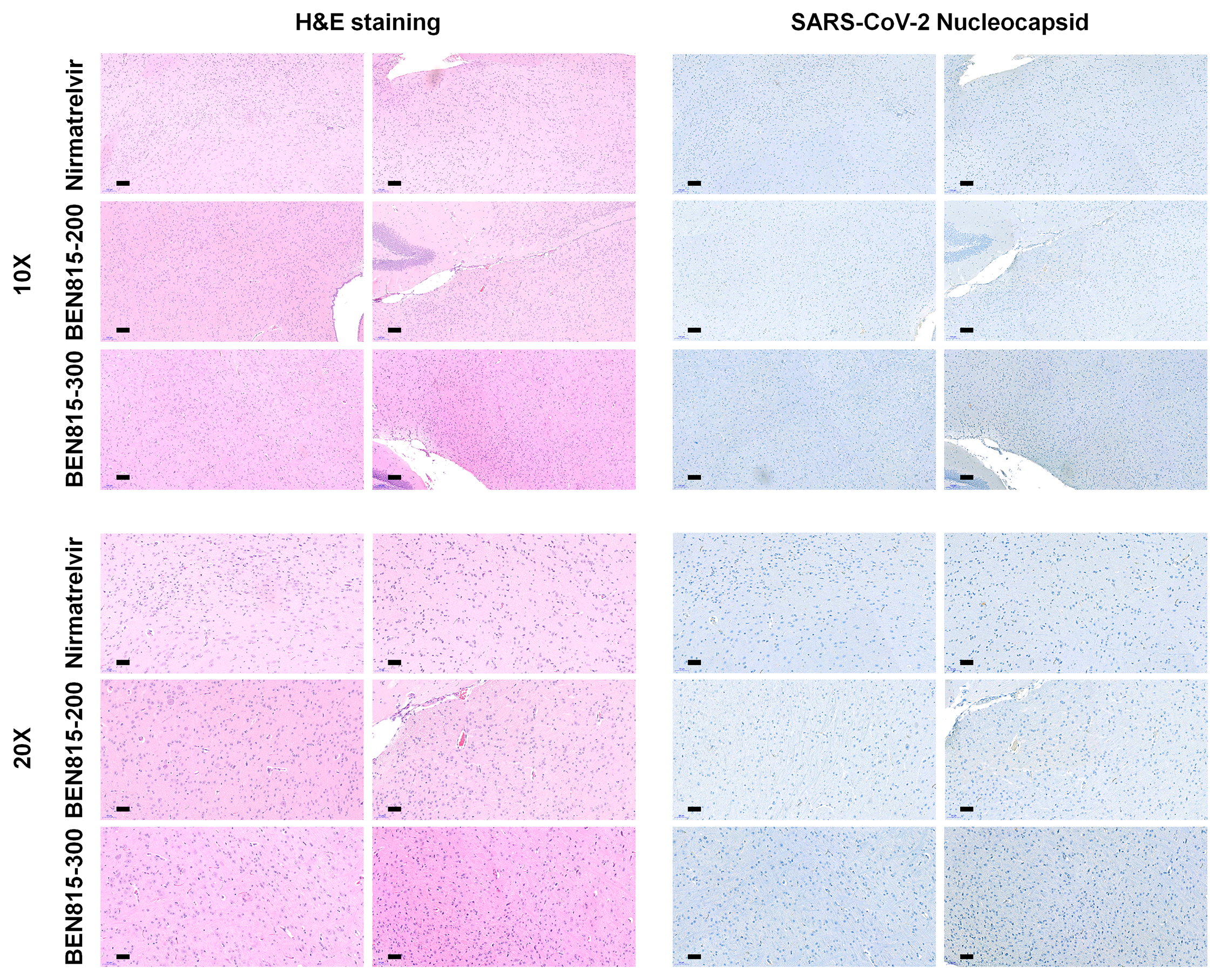

Supplement: S2 Fig — H&E and immunohistochemical staining with an anti-nucleocapsid antibody of brain tissue. Images are shown at low (10×) and high (20×) power magnifications. Scale bars represent 50 μm at 10× magnification and 100 μm at 20× magnification. (TIF) [file pone.0291537.s002.tif]
